# Using* Drosophila* Models of Amyloid Toxicity to Study Autophagy in the Pathogenesis of Alzheimer's Disease

**DOI:** 10.1155/2018/5195416

**Published:** 2018-05-20

**Authors:** Louise O'Keefe, Donna Denton

**Affiliations:** ^1^Department of Genetics and Evolution, School of Biological Sciences, The University of Adelaide, Adelaide, SA 5005, Australia; ^2^Hopwood Centre for Neurobiology, South Australian Health and Medical Research Institute, P.O. Box 11060, Adelaide, SA 5001, Australia; ^3^Centre for Cancer Biology, University of South Australia and SA Pathology, Adelaide, SA 5001, Australia

## Abstract

Autophagy is a conserved catabolic pathway that involves the engulfment of cytoplasmic components such as large protein aggregates and organelles that are delivered to the lysosome for degradation. This process is important in maintaining neuronal function and raises the possibility of a role for autophagy in neurodegenerative diseases. Alzheimer's disease (AD) is the most prevalent form of these diseases and is characterized by the accumulation of amyloid plaques in the brain which arise due to the misfolding and aggregation of toxic peptides, including amyloid beta (A*β*). There is substantial evidence from both AD patients and animal models that autophagy is dysregulated in this disease. However, it remains to be determined whether this is protective or pathogenic as there is evidence that autophagy can act to promote the degradation as well as function in the generation of toxic A*β* peptides. Understanding the molecular details of the extensive crosstalk that occurs between the autophagic and endolysosomal cellular pathways is essential for identifying the molecular details of amyloid toxicity.* Drosophila* models that express the toxic proteins that aggregate in AD have been generated and have been shown to recapitulate hallmarks of the disease. Here we focus on what is known about the role of autophagy in amyloid toxicity in AD from mammalian models and how* Drosophila* models can be used to further investigate AD pathogenesis.

## 1. Introduction

Alzheimer's disease (AD) is the most prevalent form of neurodegenerative disease characterized by deficiency in memory and cognitive functions. The predominant pathological changes of AD are the development of amyloid beta (A*β*) plaque deposits in specific brain areas and neurofibrillary tangles (NFTs) within neuronal cells, leading to the progressive loss of synapses, neuronal death, and cognitive decline [1–3]. The extracellular A*β* plaques are derived from cleavage of the amyloid precursor protein (APP). The NFTs consist of intracellular aggregates of the hyperphosphorylated microtubule-associated protein tau, mutant forms of which are also found in other neurodegenerative diseases termed tauopathies. This review will focus on the role of APP and the products arising from its proteolysis (which includes A*β*42) in AD.

While the primary mechanisms responsible for AD pathology remain to be established, there is increasing evidence for a role of the autophagy pathway in AD. Macroautophagy (referred to here as autophagy) is a conserved catabolic pathway that sequesters cytoplasmic material in a double-membrane vesicle (of nonlysosomal/vacuolar origin), the autophagosome, for delivery to the lysosome. Autophagy is induced in response to cellular stress and protects cells by eliminating dysfunctional organelles and toxic protein aggregates. Aberrant regulation of autophagy has significant adverse consequences to normal cellular functions and is associated with numerous human pathologies, including neurodegenerative diseases [4]. This review will describe the pathogenesis of AD, the conservation of components of the autophagy machinery, and their known roles in neurodegeneration. We will discuss evidence of autophagy perturbation in AD and focus on* Drosophila* as an ideal model for understanding the molecular mechanisms by which autophagy contributes to AD.

## 2. The Genetics of Alzheimer's Disease (AD)

There are two types of AD based on genetic inheritance and age of onset. Familial AD is rare, affecting approximately 1–5% of individuals that are under 65 years of age. Autosomal dominant mutations have been identified in* amyloid precursor protein* (APP) as well as* presenilin-1 (PS1)* and* presenilin-2 (PS2)* genes that encode the catalytic subunit of *γ*-secretase complex that cleaves APP to promote the generation of A*β* peptides as causative agents for familial AD [13, 14]. Sporadic, late-onset AD accounts for more than 95% of cases with both genetic and environmental factors contributing to the pathogenesis. While the genetic contribution in these patients is not fully defined, genome-wide association studies have identified several loci associated with increased AD risk in genes involved in various biological pathways including cholesterol/sterol metabolism (APOE-*ε*4), innate immunity (CR1, CD33, and TREM2), and endolysosomal and autophagy pathways (BIN1, PICALM, and CD2AP) [15, 16]. In addition, recent studies in mammalian cells further support the role of abnormal trafficking in the endolysosomal and autophagy pathways contributing to AD [17, 18].

## 3. Proteolysis of Amyloid Precursor Protein

**Figure 1:**
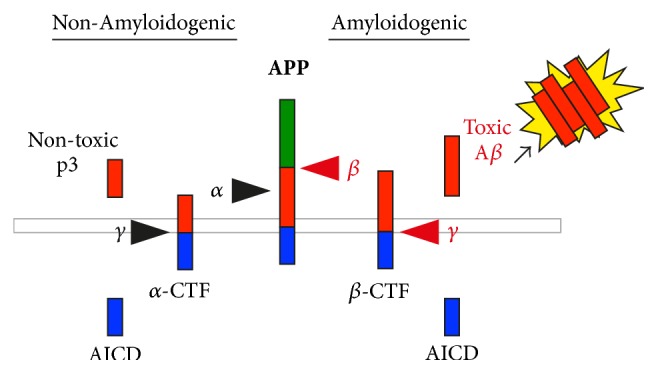
*Proteolytic processing of amyloid precursor protein*. In the nonamyloidogenic pathway, transmembrane APP is cleaved by *α*-secretase followed by *γ*-secretase generating a nontoxic P3 fragment and *α* C-terminal fragments (*α*-CTFs), thus preventing the generation of toxic A*β*. Alternatively, the amyloidogenic pathway involves sequential cleavages of APP by *β*-secretase followed by *γ*-secretase complex, thus generating toxic A*β* peptides in addition to the *β*-CTFs and amyloid precursor protein intracellular domain (AICD). The accumulation of A*β* peptides promotes oligomerisation and formation of insoluble plaques.

APP is a transmembrane protein that undergoes sequential cleavage by one of two pathways (Figure 1). The initial proteolytic cleavage of APP by either *α*-secretase (nonamyloidogenic processing) or *β*-secretase (amyloidogenic processing) produces APP-carboxy-terminal fragments (CTFs) as well as secreted APP peptides. In the nonamyloidogenic pathway, *α*-secretase (ADAM10) cleavage occurs within the A*β* region generating *α*-carboxy-terminal fragments (*α*-CTFs) and thus prevents the formation of toxic A*β* [19, 20]. The *α*-CTF is further cleaved by *γ*-secretase complex to release P3 peptide as well as an APP intracellular domain (AICD) [21]. In the amyloidogenic pathway, APP is initially cleaved by *β*-secretase 1 (beta-site amyloid precursor protein cleaving enzyme 1, BACE1) to produce *β*-carboxy-terminal fragments (*β*-CTFs). Subsequent cleavage of *β*-CTF by *γ*-secretase complex releases toxic amyloid-*β* (A*β*) peptides (Figure 1). While the processing of APP by *α*-secretase is predominantly localized to the cell surface, amyloidogenic cleavage occurs in endosomes, lysosomes, and autophagic vacuoles [22–24].

The amyloidogenic processing of APP increases the generation of A*β* that is susceptible to aggregation with other A*β* peptides accumulating into fibrils. This is commonly found in amyloid plaques in the brain (where A*β*42 aggregates are considered to be toxic) and is one of the hallmarks of AD. In addition to A*β* toxicity, the *β*-CTFs may also contribute to the pathogenesis of AD through multiple pathways [25, 26]. The AICDs of both cleavage pathways can translocate to the nucleus and induce nuclear signalling [27–29]. However, the principle physiological functions of APP remain largely undetermined. The proposed role for APP acting as a cell surface receptor or as a ligand, such as transcriptional regulation and/or synaptic functioning, requires further* in vivo* characterization [30]. While the generation of extracellular A*β* plaques is central to the hypothesis of amyloid as the causative agent in AD [31], additional factors have been identified which may contribute to the onset and/or progression of AD with dysregulation of autophagy thought to be an early event. Despite advances in the understanding of AD pathogenesis, further studies are required to understand the molecular mechanism by which autophagy contributes to disease pathogenesis. In addition, the consequence of A*β* as well as other products from APP processing on other cellular processes including autophagy needs further investigation.

## 4. Autophagy Molecular Machinery

**Figure 2:**
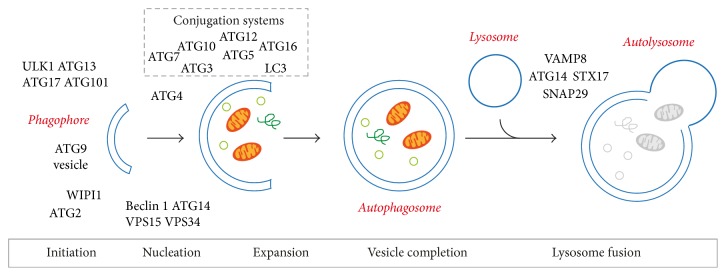
*Schematic representation of the autophagy pathway and the regulatory machinery*. The multiple steps of autophagy can be divided into initiation, nucleation, expansion, vesicle completion, and lysosome fusion. Several ATG proteins form distinct complexes that function in different stages of autophagy. The ULK1/ATG1 complex (consisting of ULK1, ATG13, ATG17, and ATG101) is responsible for the initiation of autophagy. The class III phosphatidylinositol 3-kinase (PI3K) complex (BECN1, VPS34, VPS15, and ATG14), ATG9, and ATG2-WIPI complex nucleate and assemble the membrane to form the double-membrane phagophore. The LC3 and ATG12 conjugation systems can be involved the formation of the autophagosome. Once completed, the autophagosome fuses with the lysosome where the enclosed components are degraded by lysosomal enzymes.

Autophagy is a highly conserved catabolic pathway that degrades/recycles cytoplasmic material such as large protein aggregates and organelles. The cytoplasmic components are engulfed by a double-membrane vesicle, the autophagosome, for delivery to the lysosomes for degradation (Figure 2). Autophagy has essential functions in normal development, cell growth, metabolism, cell death, infection, and immunity [32–34]. It also acts to protect cells by eliminating toxic protein aggregates, unwanted cellular contents, dysfunctional organelles, and invading pathogens. Under growth-promoting conditions, low basal rates of autophagy are required to maintain cellular homeostasis. In response to extracellular and intracellular stresses, such as nutrient limitation, intracellular metabolic stress, organelle damage, and infection, high levels of autophagy are induced to recycle cytoplasmic material to maintain vital cellular processes [35]. The tightly coordinated multistep process of autophagy is regulated by a number of distinct autophagy-related (ATG) gene products that assemble into specific complexes [36]. Many of these components are evolutionarily conserved from yeast to mammals, including in* Drosophila *(Table 1). The autophagy process/complexes can be functionally divided into (i) initiation, (ii) autophagosome nucleation, (iii) expansion and vesicle completion, and (iv) lysosome fusion [37–40] (Figure 2).

### 4.1. Initiation and Nucleation

Autophagy is initiated by the formation of a double-membrane structure called the phagophore (also called the isolation membrane) that further expands to encapsulate part of the cytoplasm into the autophagosome [41]. A key early step in autophagy induction requires the activity of the ULK1 (Atg1 in* Drosophila*) kinase complex, comprising ULK1/Atg1, ATG13, FIP200/ATG17, and ATG101. The activity of this complex is regulated in response to stress signals [42]. Nucleation from the phagophore (isolation membrane) requires active ULK1 kinase complexes for the recruitment of class III phosphatidylinositol 3-kinase (PI3K) complex. This complex consists of VPS34, VPS15, ATG14, and Beclin 1 (Atg6 in* Drosophila*) to generate phosphatidylinositol 3-phosphate (PI3P) required for vesicle nucleation.

### 4.2. Expansion and Vesicle Completion

The expansion and completion to form the autophagosome requires two ubiquitin-like conjugation systems: the Atg8/LC3-lipid phosphatidylethanolamine (PE) and the ATG12-ATG5 systems [37, 43, 44]. There are six Atg8 family members in mammals, including LC3A, LC3B, LC3C, and GABARAP proteins [45] and two in* Drosophila* with Atg8a shown to be essential for autophagy [46]. Prior to lipidation, LC3/Atg8 is cleaved to produce a C-terminal glycine residue (LC3-I form) by the cysteine protease, ATG4. This enables the conjugation of LC3 to PE mediated by ATG7 (E1-like enzyme) and ATG3 (E2-like enzyme). In the ATG12-ATG5 conjugation system, ATG7 and ATG10 (E1- and E2-like enzymes, respectively) mediate the conjugation of ATG12 to ATG5, which associates with ATG16. To enable phagophore expansion a supply of lipid bilayers is required and is thought to involve the transmembrane protein ATG9; however its exact function remains unclear. Membrane closure is thought to involve ATG2, in combination with WIPI1/Atg18, to regulate autophagosome formation [47].

### 4.3. Lysosomal Fusion

The final step in autophagy is fusion of the autophagosome with the lysosome to form an autolysosome [37]. Lysosomes are specialized organelles that function to break down extracellular materials and recycle cellular components from the secretory, endocytic, autophagic, and phagocytic pathways [48]. The lysosome contains hydrolytic enzymes required for degradation, including cathepsin proteases that are activated by the acidic pH within the lysosome generated by proton-pumping vacuolar H+ ATPase (v-ATPase) [49]. The fusion between autophagosome and lysosome requires the soluble N-ethylmaleimide-sensitive factor activating protein receptor (SNARE) complex consisting of syntaxin 17 (STX17) and synaptosomal-associated protein 29 (SNAP29). This complex forms on autophagosomes to promote tethering with vesicle-associated membrane protein 8 (VAMP8) on lysosomes, resulting in fusion to form autolysosomes [50].

### 4.4. Amphisome Formation

Instead of fusing with a lysosome, an autophagosome can also fuse with a late endosome/multivesicular body to form an amphisome [51]. Amphisomes contain markers of both autophagosomes (lipidated LC3) and endosomes (RAB5, RAB7, and RAB11) [52, 53]. Members of the endosomal sorting complex required for transport (ESCRT) complex are required during endocytosis as well as for later endosomal maturation and amphisome formation during autophagy [54].

### 4.5. Selective Autophagy

The targeting of cytoplasmic material to the autophagosome can also occur in a specific manner, by recognizing selective substrates. These can include, for example, damaged mitochondria (mitophagy), excess peroxisomes (pexophagy), and aggregate-prone proteins, including those causing many neurodegenerative conditions (aggrephagy) [38]. The selection of autophagic cargo can be determined by cargo receptors that interact with LC3 family member proteins on the membrane [55]. The multifunctional scaffold protein SQSTM1/p62 (known as Ref(2)P in* Drosophila*) binds ubiquitinated proteins and acts as a cargo receptor by binding LC3/Atg8 targeting ubiquitinated proteins for degradation by autophagy [56]. The type of ubiquitin linkages on the substrate can lead to different functional outcomes. The most common ubiquitin linkage tags proteins for degradation of the ubiquitin-proteasome system, whereas other linkages can direct nonproteasomal fates. There are a growing number of identified cargo receptors that bind specific substrates that are tagged with ubiquitin chains. Thus, the receptors serve as a link between ubiquitinated cargo and the autophagy pathway to enable the selective incorporation of the cargo into autophagosomes.

## 5. Role for Autophagy in Maintaining Neuronal Homeostasis

Multiple upstream signalling pathways regulate autophagy induction with nutrient deprivation, one of the most well characterized signals. The target of rapamycin (TOR) kinase is a central mediator in regulating the response to nutrients and growth signalling and forms a multisubunit complex, mTORC1 [57]. In the presence of growth signals, mTORC1 is activated, thus preventing autophagy by inhibiting ULK1/Atg1 kinase. Under growth-limiting conditions, mTORC1 is no longer active in enabling autophagy induction by activation of ULK1/Atg1 [58, 59]. Numerous studies link alterations of mTOR pathway to age-dependent cognitive decline and to pathogenesis of Alzheimer disease (AD) [60], highlighting the importance of maintaining physiological levels of autophagy to promote neuronal health.

Several nervous system-specific conditional knockout mouse models of autophagy pathway genes have highlighted the importance of autophagy in maintaining the normal functions and homeostasis of the nervous system. The conditional deletion of* Atg5* and* Atg7* in neuronal precursor cells results in autophagy deficiency, accompanied by the accumulation of intraneuronal aggregates in neurons resulting in neuronal loss and neurodegeneration [61–63]. The accumulation of these aggregates in otherwise normal mice suggests that autophagy plays a key role in removing aggregate-prone proteins. Other mouse models of autophagy deficiency, including conditional knockout for* FIP200* and* Wipi4*, as well as* Ulk1*/2 double knockout [64–66], similarly show reduced survival and early-onset, progressive neurodegeneration across broad areas of the brain. However, each model presents variations in the pathology observed which may be due to the specific stage of autophagy that is disrupted, as well as any potential autophagy independent gene functions.

Reduced function of conserved autophagy genes in* Drosophila* also results in neurodegenerative phenotypes (Table 1). Initiation of autophagy requires* Atg17/Fip200*, and reduced expression in adult flies resulted in a climbing defect as well as decreased survival [6].* Atg5 *null flies displayed mobility defects [7], and decreased* Atg16 *resulted in climbing defects and decreased survival [11].* Atg7 *mutants show a shortened lifespan as well as accumulation of aggregated ubiquitin-positive lesions in neuronal cells [9] while* Atg8a* mutants that are viable show decreased lifespan [10]. Taken together, these studies demonstrate the critical and conserved role of autophagy in neuronal homeostasis with the impaired clearance by autophagy likely to be a key factor in the accumulation of toxic peptides in the neurons.

## 6. Autophagy in Alzheimer's Disease

A hallmark of AD and other neurodegenerative diseases is the accumulation of large protein aggregates/inclusions and defective organelles. Autophagy is an essential degradation pathway involved in the clearance of abnormal protein aggregates as well as maintaining protein homeostasis in neuronal cells [67]. There is substantial evidence from both AD patients as well as animal models for the dysregulation of autophagy in this disease. Current findings suggest that impairment of the autophagy pathway leads to defects in the clearance of protein aggregates which is likely to occur early in the pathogenic process, before plaque formation or NFTs deposition [68–70]. However the role of autophagy in AD (in particular which stage is affected) and its alteration during disease progression in neurons is complex and remains largely unclear. Alterations to autophagy have also been identified in other neurodegenerative diseases, including Parkinson's disease and Huntington's disease [71]. There is also evidence for mitophagy in these diseases; however, that is outside the focus of this review and has been reviewed elsewhere [72]. In healthy neurons, autophagy is constitutively active and highly efficient, with low levels of autophagosomes detected [73]. Early observations revealed the accumulation of abnormal subcellular vesicles in the dystrophic or swollen neurites in AD patient brains [74]. Further evidence for disruption to autophagy flux in AD was revealed by the identification of autophagosomes and other immature autophagic vesicles that accumulated in dystrophic neurites in AD brains [68]. While clinical data has identified defects in autophagosomal biogenesis, whether this is pathogenic or a consequence of earlier defects is still controversial. Also, there is evidence that autophagy may not only act to promote the degradation of A*β* but may also be involved in its generation [8, 75].

To aid in understanding the role of autophagy in AD, animal models have provided a tool for* in vivo* studies. A number of transgenic mouse models have been generated based on the genetic pathways disrupted in AD [76]. In an APP/PS1 transgenic mouse model that contains human transgenes for APP and PS1, both of which are carrying human disease mutations, neuronal autophagy is detected in the brain before the appearance of A*β* plaques [22]. Consequently, autophagosomes and late autophagic vacuoles/intracellular trafficking vesicles accumulate in dystrophic dendrites, suggesting impaired maturation of autophagosomes to lysosomes [22]. Similarly, in another study young (4- to 6-month-old) APP/PS1 mice accumulated abnormal immature autophagosomes in axons of hippocampus neurons before neuronal loss [77]. The localization of both APP and PS1 to autophagic vacuoles suggests that A*β* may be generated during autophagy [22, 78]. This indicates that accumulation of autophagic vacuoles/intracellular trafficking vesicles may be a source of A*β* production contributing to AD progression.

Altering the level of autophagy has also been examined in AD models. APP transgenic mice with* Atg7* deletion showed a reduction in A*β* extracellular secretion and plaque formation [79, 80]. This block in A*β* secretion resulted in an accumulation of intracellular A*β* and enhanced neurodegeneration was observed. An increase in the level of autophagy by rapamycin inhibition of mTOR in APP transgenic mice reduced A*β* levels and prevented AD-like cognitive deficits [81]. These findings suggest that autophagy may function in either degradation or secretion in A*β* and supports a role for autophagy in limiting the accumulation of toxic A*β*.

There is further evidence from animal models that basal autophagy is beneficial for decreasing the pathology in AD. In the APP mouse model of AD, heterozygous deletion of* BECN1* decreases neuronal autophagy and increases the accumulation of both intraneuronal and extracellular A*β* deposits followed by neurodegeneration [82]. In support of this, reduced levels of* Beclin 1/BECN1* have been detected in the brains of patients with severe AD [82]. Consistent with this, a mouse knockin of a Beclin 1 gain of function mutation resulted in constitutively active autophagy and, when combined with an AD mouse model, showed reduced A*β* accumulation, prevented cognitive decline, and restored survival [83]. This suggests that in AD, BECN1 induced autophagy contributes to reduction in levels of A*β* peptides/aggregates. In an alternative approach, aged (7-month-old) APP/PS1 transgenic mice were transfected with miR-124 lentiviral vector that downregulates BACE1 [84]. These mice also showed increased Beclin 1 with alleviation of AD pathology but surprisingly they had decreased expression of other autophagy markers. This suggests that Beclin 1 may not be acting via the autophagic pathway in this system and may have other roles such as in the PtdIns 3-kinase complex (Rubicon-UVRAG-Beclin 1-hVps34-hVps15) that localizes to the late endosome/lysosome and inhibits autophagy [85]. Together these data highlight the need for comprehensive* in vivo* analyses to dissect the role of individual autophagy genes in AD pathogenesis.

## 7. Crosstalk between Autophagy and the Endolysosomal System in AD

The subcellular distribution of APP plays a key role in A*β* production and occurs within the autophagy and endolysosomal systems [86, 87]. The early endosome is the site of colocalization of APP and BACE1 promoting the proteolytic cleavage of APP [88, 89]. Indeed, endosomal pathology is one of the earliest defects observed in AD [90, 91]. Altered levels of the endosomal small GTPase, Rab5, precede A*β* deposition [91], and A*β* colocalizes in Rab5 endosomes in neurons from AD brain [23]. More recently, expression of a dominant negative Rab5 mutant was shown to reduce APP-induced axonal blockages in both cultured neurons and an* in vivo Drosophila* model [92]. Genome-wide association studies identified mutations in endosomal genes including* BIN1*,* CD2AP*, and* PICALM*, which supports the involvement of the endosomal network in processing and trafficking of APP proteolytic fragments [15].* Drosophila *homologues of these genes show interactions with increased tau expression [17, 93, 94] but they have not been tested with respect to amyloid pathology.

The metabolism of APP in endolysosomal and autophagy networks is consistent with crosstalk between these pathways. Autophagic and lysosomal genes are coordinately regulated by a complex transcriptional program mediated by Transcription Factor EB (TFEB) [95]. TFEB levels have been found to be decreased in brains of Alzheimer's patients [96] while an increase in TFEB expression has been shown to be protective for A*β*-induced pathogenesis [97]. Similarly, in an APP/PS1 mouse model, the overexpression of TFEB increases lysosome biogenesis and reduces A*β* levels [98]. In* Drosophila *there is a single TFEB orthologue,* Mitf*, which has been shown to have a role in regulation of the v-ATPase proton pump as well as other components of the lysosomal-autophagic pathway to promote clearance of protein aggregates [99, 100].

As both endocytic and autophagic pathways lead to the lysosome, it is not surprising that aberrant lysosomal function contributes to AD pathogenesis. Defective lysosomal membrane integrity has been detected in AD patients suggesting dysfunction [101]. Increased expression levels of lysosomal proteases in the early phase of AD patients have also been reported [102]; it is likely that this increased lysosomal function is in response to increased pathogenic load. The AD-associated risk factor gene Apolipoprotein E4 (ApoE4) also affects lysosomal function. Transgenic mice that overexpress ApoE4 accumulate A*β*42 in lysosomes and there is death of neurons in the hippocampus [103]. Also in Neuro-2a cells, ApoE4 can affect lysosomal membrane permeabilization causing the release of proteolytic enzymes that can mediate cell death [104]. Further support for the function of lysosomes in AD was highlighted by the role of PS1 in the assembly of the v-ATPase pump in the lysosomal membrane, thus promoting acidification and contributing to autophagy degradation in a *γ*-secretase-independent way [105]. An alternative report suggested that the lysosome dysfunction resulting from loss of PS1 could be attributed to alterations in lysosomal calcium storage [106]. Increased or sustained activation of Glycogen synthase kinase-3 also affects lysosome acidification and has been shown to affect the autophagic degradation of APP [107, 108]. In addition, consideration needs to be given to the physiology of neuronal cells where retrograde transport of distally located autophagic vacuoles (mostly amphisomes) is required before any fusion can occur with lysosomes that are located in the soma [109].

These findings and others, including cell culture studies not described here, clearly establish autophagic and endolysosomal dysfunction in AD. Using model organisms to gain an understanding of the exact contribution of these pathways to the pathogenesis of AD will be a priority to enable the development of specific therapeutic interventions that do not affect other essential cellular processes.

## 8. Advantages of Using* Drosophila* to Model Amyloid Pathology

**Figure 3:**
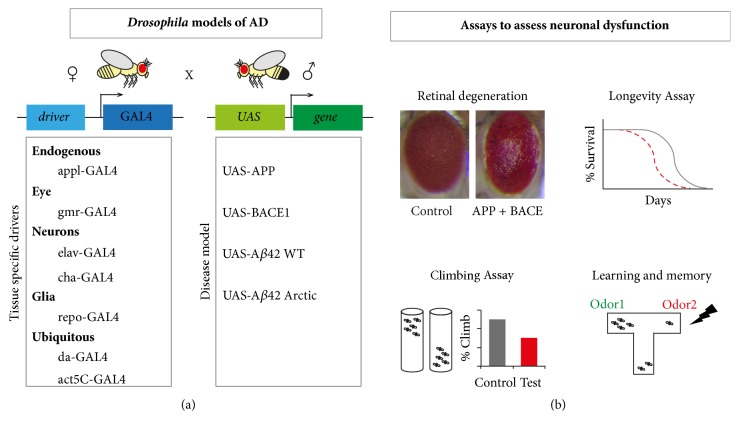
*Drosophila models of AD and assays for neurodegeneration*. (a)* Drosophila* models of AD. The GAL4/UAS system is routinely used in* Drosophila* to drive expression of a gene of interest [12]. There are ubiquitous or tissue-specific enhancers that drive expression of GAL4. By crossing lines containing the driver-GAL4 to the UAS-gene of interest, the progeny will result in ectopic expression. Eye, neuronal, glial, or ubiquitous drivers are used to express A*β* or APP and BACE1 transgenes resulting in specific phenotypes. These can be assessed for neural degeneration and dysfunction. (b) Assays to assess neuronal dysfunction. Using the eye-specific driver GMR-GAL4, APP + BACE1 can be expressed during eye development and the adult eye disruption can be observed. The degenerative eye shows disruption of ommatidial structure, reduced size, and loss of pigmentation. This is a useful system to screen for modifiers of APP + BACE1 toxicity. Lifespan analyses can be performed using neuronal, glial, or ubiquitous cell type driver lines and the effect of genetic modifiers on the longevity of APP + BACE1 flies can also be monitored. Climbing assays can be used to examine locomotor deficits that are known to degenerate with age. Flies are tapped to the bottom of a measuring cylinder and the number of flies that can climb above a certain height is recorded. Also relevant for studies in AD are assays for learning and memory such as odour preference teamed with an electrical shock treatment.

More than 77% of human disease genes listed on the OMIM database have an orthologue in* Drosophila*, confirming their utility as a model for human genetic diseases [110]. In addition, it is possible to avoid complications that could arise from redundancy as there is often a single gene in* Drosophila *compared with multiple genes in mammalian systems as is the case for APP (Table 2). Knockdown and ectopic expression constructs are readily available in* Drosophila* for most genes and the genetic toolkit available for analyses is constantly being developed and refined [111]. Ectopic expression via the GAL4/UAS system is used most frequently where various tissue-specific “drivers” (i.e., gene-specific promoter regions upstream of a GAL4 transcriptional activation domain) give particular patterns of expression. Driver lines most useful for studies of molecular mechanisms of AD include the endogenous APPL promoter (appl-GAL4), the eye driver (gmr-GAL4), the neuronal driver (elav-GAL4), and ones that express specifically in cholinergic neurons (cha-gal4), glial cells (repo-GAL4), or ubiquitously (da-GAL4 or actin5C-GAL4) (Figure 3(a)) [12]. Inducible expression systems are also available (e.g., GeneSwitch) which allow for studies where the timing of transgene expression can be regulated more precisely [112].

Ectopic expression of human sequences encoding full length APP (with or without BACE1) or A*β*1–42 peptides (wild-type or mutant) in transgenes under UAS control gives rise to neuronal dysfunction which can be measured as retinal degeneration, locomotor defects, decreased longevity, learning and memory defects, and alterations to various cell biological markers [113] (Figure 3). These have been used as the basis for genetic and/or pharmacological screening [114–117]. Other novel approaches to ameliorating AD symptoms in* Drosophila* models include immunotherapy and photodynamics [118, 119]. In addition, the contribution of nonneuronal cell types to AD disease progression is well established. Glial cells have been shown to clear neurotoxic A*β* peptides in the adult* Drosophila* brain through a Draper/STAT92E/JNK cascade that may be coupled to protein clearance pathways such as autophagy [120]. The genetic systems available in* Drosophila *also allow for elegant approaches for understanding the complex interactions that occur between neurons and glia that could contribute to AD [121].

## 9. *Drosophila* Models for Amyloid Toxicity

Components of APP proteolysis are conserved in* Drosophila* (see Table 2). Although there is limited sequence conservation across the A*β*42 region, it has been shown that neuronal dBACE like enzyme activity can lead to cleavage of the APP-like (APPL) protein in* Drosophila* where the resultant peptide gives rise to neurodegenerative phenotypes that are accompanied by A*β*-like deposits [122]. Processing of APPL gives rise to the same types of cleavage fragments shown for human APP in Figure 1 including small membrane bound intracellular CTFs and neurotoxic A*β*-like peptides, and these have been shown to be expressed throughout the nervous system during development [123]. Given that APPL is conserved throughout evolution suggests that it does have important functions, some of which have been uncovered including its role in neuronal outgrowth and synapse formation, regulation of the circadian clock, and providing neuroprotection in models for AD as well as other neurodegenerative diseases [123–125].

Despite the conservation of endogenous APP processing and function in* Drosophila*, disease models have predominantly been generated based on ectopic expression of human counterparts based on the mutations identified in genetic pathways involved in AD (Table 2) (Figure 3). Various model systems have been developed whereby the human gene products are ectopically expressed in* Drosophila.* Many studies have determined the effects of expressing the A*β*42 toxic peptide directly and it has been shown to give rise to age-dependent neurodegenerative phenotypes that are accompanied by significant disruption to the correct functioning of the autophagic-lysosomal system [126]. It was shown that A*β*42 carrying the “Arctic” APP human disease mutation (E22G) has more severe effects as it is thought to increase the rate of A*β*42 aggregation [127, 128]. However these A*β*42 expression constructs require the inclusion of signal sequences from unrelated genes to ensure their secretion and it has been shown that, at least in some cases, these can give alternative effects [129]. Nonetheless, A*β*42 is localized within endosomes and has been proposed to be the cellular source of pathogenic A*β*42 [8]. The presence of A*β*40 was also observed but found not to correlate with toxicity. Similarly comparison of A*β*40 with A*β*42 by others has also shown differential effects in memory testing [130]. In addition ectopic expression of A*β*43 was tested separately and found to be neurotoxic, potentially by acting to prime the formation of amyloid aggregates [131].

Ectopic expression of the full length (695 amino acid) APP is also used in* Drosophila *models for AD where again both wild-type and disease associated mutations have been investigated. Wild-type human APP expressed in combination with ectopic human BACE1 enzyme gives effective processing of APP and leads to neuropathology [132]. Synaptic abnormalities have also been reported when APP and BACE1 are coexpressed specifically in neuronal cells [133]. Interestingly, it has been shown that equivalent amounts of A*β*42 peptide produced from processing of APP (when it is expressed together with BACE1) give stronger effects* in vivo* than A*β*42 peptide expressed directly as the secreted form [134]. This suggests that incorporating the findings from APP and BACE1 ectopic expression models will contribute significantly to the understanding of the molecular pathogenic mechanisms of the proteolytic products of APP.

## 10. Role for Autophagy in* Drosophila* Models for AD

Similar to mammalian systems there is accumulating evidence for a role of autophagy in the pathogenesis of* Drosophila *models for AD. Amyloid toxicity models tested to date have concentrated on those ectopically expressing the A*β*42 peptide (Table 2). Increased basal autophagy by various methods in these models suppresses ectopic A*β*42 induced phenotypes [135, 136]. Specific components of the autophagy pathway have also been investigated by genetic modification analyses in these A*β*42 models. Decreased expression of* Atg1* or* Atg18* was found to enhance the neurotoxic effect in flies expressing A*β*42, also supporting a protective role for autophagy [5]. However, contrary to this, the knockdown of* Atg5* or* Atg12* was shown to decrease accumulation of A*β*42 [8]. These findings suggest that there is a complex role for components of the autophagic pathway in AD which may be attributed to the particular stage of the process and/or correlate with timing of disease progression. Together they highlight the need for a comprehensive genetic dissection of the autophagy pathway to determine its contribution to AD.

## 11. Ageing, Autophagy, and AD

Age is the most prominent risk factor in the development of AD. Age-related dysfunction of autophagy may play a causative role in the onset and progression of AD. It has been suggested that the neuronal autophagy-lysosomal system may shift from a functional and protective state to a pathological and deleterious state either during brain ageing or via A*β*42 neurotoxicity [137]. In support of this there is also an age-related decline in clearance of A*β*42 via the X-box protein 1 [138]. An aged onset model has been developed in* Drosophila* where human APP and human BACE1 are expressed at low levels during development followed by increased expression throughout adulthood [139]. This type of model will enable* in vivo* studies in* Drosophila* to more closely represent disease progression as it occurs in humans.* Drosophila* is also an excellent model to dissect the molecular mechanisms of ageing that are relevant for AD related neuronal dysfunction [140].

## 12. Conclusions and Future Directions

The contribution of autophagy to AD has been controversial. In particular, it remains to be determined whether autophagy plays a causative or a protective role in AD or whether autophagy defects are a consequence of disease progression. The detection of aberrant autophagy alone is not sufficient to support a causative role, and further detailed molecular analysis is required. However, there is clear evidence to suggest that autophagy is involved in AD pathophysiology. With therapeutic intervention based on modulating autophagy, it will be critical to understand the role of autophagy in the different stages of the disease as well as defining the molecular mechanisms underlying autophagy dysfunction in AD. While the strongest evidence for the contribution of dysfunctional autophagy to AD comes from* in vivo* studies,* in vitro* cell studies have contributed to the understanding of autophagy defects in AD.

Disruption to autophagy could occur at different steps in the pathway from initiation, elongation, cargo selection, lysosomal fusion, and degradation. This may result in altered autophagic flux, with accumulation of autophagosomes, autolysosomes and/or amphisomes, and lysosomal defects that may present as different pathological outcomes. In addition, there is a tissue-specific requirement for distinct components of the autophagic machinery as well as autophagy independent functions of a number of* Atg* genes [46, 141]. Given the controversy as to the protective and/or pathogenic role of autophagy in AD, using* Drosophila* models to dissect out the contribution of the different steps will provide important information about the origin of dysfunctional autophagic processes in AD.

Alzheimer's disease pathology is remarkably complex and human genetic mutations have highlighted alterations to amyloid processing as a primary event that gives rise to neuronal toxicity. Autophagy as part of a cellular clearance mechanism has been shown to play a prominent role in disease progression but its functional contribution to neurotoxicity and/or neuroprotection has not been fully defined. In addition some clues have emerged as to the role of nonneuronal cells, in particular glial cells and their interactions with neuronal cells that can affect neuronal function. Using the genetic platform provided by* Drosophila*, these pathways can be fully dissected and cellular mechanisms of neuronal dysfunction identified. This could include a multigenic approach where more than one candidate can be tested for their effects on APP processing and disease progression. In addition, given that ageing is the most prominent risk factor in AD, the time-frame that would be required for determining efficacies of drugs in humans is not feasible. With the development of technology that can detect amyloid in the blood as an early biomarker for Alzheimer's disease [142, 143], this now provides the opportunity for early intervention and there is a pressing need for identifying new therapeutic compounds. By understanding the role of autophagy in progression/prognosis, this will provide potential novel ways to treat AD and/or provide prognostic biomarkers of disease. Again* Drosophila* presents as an ideal system where specific autophagic mechanisms could be targeted for the development of novel therapies for early intervention in AD.

## Figures and Tables

**Table 1:** Conserved *Autophagy-related* genes in *Drosophila*, neurodegenerative phenotypes, and/or modification of AD models.

	Human gene	*Drosophila* gene	Neurodegenerative phenotype in *Drosophila*	Modification of AD model in *Drosophila*
Initiation	ULK1/ATG1	Atg1	Decreased lifespan and climbing defect [6]	Deficiency line with decreased *Atg1* reduced lifespan of A*β*42 expressing flies [5]
	ULK2			
	ATG13	Atg13		
	FIP200/RB1CC1	Atg17		
	ATG101	Atg101		

Nucleation	BECN1	Atg6		
	ATG14	Atg14		
	PIK3R4/VPS15	Vps15/ird1		
	PIK3C3/VPS34	Vps34/Pi3K59F		

Conjugation systems	ATG3	Atg3/Aut1		

Conjugation systems	ATG4A	Atg4a		
	ATG4B			
	ATG4C	Atg4b		
	ATG4D			

Conjugation systems	ATG5	Atg5	Climbing defect [7]	Decreased *Atg5* reduced A*β*42 accumulation [8]

Conjugation systems	ATG7	Atg7	Decreased lifespan and climbing defect [9]	

Conjugation systems	MAP1LC3A	Atg8a	Reduced lifespan [10]	
	MAP1LC3B			
	MAP1LC3C			
	GABARAP			
	GABARAPL1	Atg8b		
	GABARAPL2			

Conjugation systems	ATG10	Atg10		

Conjugation systems	ATG12	Atg12		Decreased *Atg12* reduced A*β*42 accumulation [8]

Conjugation systems	ATG16L1	Atg16	Decreased lifespan and climbing defect [11]	

ATG9 trafficking system	ATG9A	Atg9		
	ATG9B			

ATG9 trafficking system	ATG2A	Atg2		
	ATG2B			

ATG9 trafficking system	WIPI1			
	WIPI2	Atg18a		Deficiency line with decreased *Atg18a* reduced lifespan of A*β*42 expressing flies [5]
	WDR45B/			
	WIPI3	Atg18b		
	WDR45/WIPI4			

**Table 2:** The human genes that function in APP proteolysis and their *Drosophila* orthologues.

Human gene	*Drosophila* gene	Functions
Amyloid precursor protein (APP)	Appl	APP is an integral membrane protein containing an A*β*-like region that is cleaved by BACE1.
Amyloid precursor-like proteins (APLP1 and APLP2)		Sequence divergence at the internal A*β* site of APLP1 and APLP2 prevents cleavage by BACE1. The principal functions of APLP1 and APLP2 remain unknown.

Presenilin 1	Presenilin	The catalytic subunit of the *γ*-secretase enzyme complex, also required for lysosomal acidification.
Presenilin 2		Component of *γ*-secretase complex.

ADAM 10	Kuzbanian	A neuronal *α*-secretase that cleaves APP at the plasma membrane via nonamyloidogenic processing.

BACE1	Bace	*β*-secretase enzyme activity cleaves APP in early endosome and promotes amyloidogenic processing with A*β* production.
BACE2		*β*-secretase related to BACE1 that is thought to contribute to Alzheimer's disease.
